# Pyrroloquinoline Quinone Resists Denervation-Induced Skeletal Muscle Atrophy by Activating PGC-1α and Integrating Mitochondrial Electron Transport Chain Complexes

**DOI:** 10.1371/journal.pone.0143600

**Published:** 2015-12-08

**Authors:** Yung-Ting Kuo, Ping-Hsiao Shih, Shu-Huei Kao, Geng-Chang Yeh, Horng-Mo Lee

**Affiliations:** 1 Graduate Institute of Medical Sciences, College of Medicine, Taipei Medical University, Taipei, Taiwan; 2 Department of Pediatrics, School of Medicine, College of Medicine, Taipei Medical University, Taipei, Taiwan; 3 Department of Pediatrics, Shuang Ho Hospital, Taipei Medical University, Taipei, Taiwan; 4 School of Medical Laboratory Science and Biotechnology, College of Medical Science and Technology, Taipei Medical University, Taipei, Taiwan; University of Texas Health Science Center at San Antonio, UNITED STATES

## Abstract

Denervation-mediated skeletal muscle atrophy results from the loss of electric stimulation and leads to protein degradation, which is critically regulated by the well-confirmed transcriptional co-activator peroxisome proliferator co-activator 1 alpha (PGC-1α). No adequate treatments of muscle wasting are available. Pyrroloquinoline quinone (PQQ), a naturally occurring antioxidant component with multiple functions including mitochondrial modulation, demonstrates the ability to protect against muscle dysfunction. However, it remains unclear whether PQQ enhances PGC-1α activation and resists skeletal muscle atrophy in mice subjected to a denervation operation. This work investigates the expression of PGC-1α and mitochondrial function in the skeletal muscle of denervated mice administered PQQ. The C57BL6/J mouse was subjected to a hindlimb sciatic axotomy. A PQQ-containing ALZET® osmotic pump (equivalent to 4.5 mg/day/kg b.w.) was implanted subcutaneously into the right lower abdomen of the mouse. In the time course study, the mouse was sacrificed and the gastrocnemius muscle was prepared for further myopathological staining, energy metabolism analysis, western blotting, and real-time quantitative PCR studies. We observed that PQQ administration abolished the denervation-induced decrease in muscle mass and reduced mitochondrial activities, as evidenced by the reduced fiber size and the decreased expression of cytochrome *c* oxidase and NADH-tetrazolium reductase. Bioenergetic analysis demonstrated that PQQ reprogrammed the denervation-induced increase in the mitochondrial oxygen consumption rate (OCR) and led to an increase in the extracellular acidification rate (ECAR), a measurement of the glycolytic metabolism. The protein levels of PGC-1α and the electron transport chain (ETC) complexes were also increased by treatment with PQQ. Furthermore, PQQ administration highly enhanced the expression of oxidative fibers and maintained the type II glycolytic fibers. This pre-clinical *in vivo* study suggests that PQQ may provide a potent therapeutic benefit for the treatment of denervation-induced atrophy by activating PGC-1α and maintaining the mitochondrial ETC complex in skeletal muscles.

## Introduction

Skeletal muscle has critical physiological functions including energy expenditure, metabolism, and physical strength. Skeletal muscles are divided into two isoforms based on their metabolism: type I fibers are more reddish, with a slower contractile speed and greater fatigue resistance, and with greater mitochondrial content, favoring oxidative respiration. On the other hand, type II fibers are whitish with a faster contractile speed and lower mitochondrial content, and more easily become fatigued [1, 2]. Healthy muscle preserves a balance between protein biosynthesis and degradation. Decreased muscle mass or atrophy represent the acceleration of protein degradation induced by various physiological challenges such as chronic and acute diseases (diabetes and trauma), disuse conditions (denervation and microgravity), and progressive aging or sarcopenia [3]. Denervation of peripheral motor nerves results in dysfunction of skeletal muscle contractility [4]. These changes include a rapid loss of muscle mass and mitochondrial function during the first week after denervation [5]. During long-term denervation, skeletal muscle undergoes atrophy resulting from the loss of neural input. Skeletal muscle atrophy is followed by an increase in fibrous and adipose connective tissue and subsequently the loss of muscle function [6].

Cellular energy metabolism is divided primarily by mitochondrial oxidative phosphorylation (OXPHOS) and glycolysis. Mitochondria play a central role in muscle, modulating the balance between biogenesis and degradation, which are regulated by environmental stimulation and thus transcriptionally control the down-stream expression of nuclear and mitochondrial genes [7]. Furthermore, while denervation-induced muscle atrophy has been reported to be involved in the mitochondrial reactive oxygen species (ROS) burst [8], the influence of denervation on muscle energy metabolism has received less discussion. The transcriptional coactivator peroxisome proliferator-activated receptor γ coactivator-1 α (PGC-1α) is one of the best-recognized regulators of mitochondrial biogenesis [1, 9]. Recent studies revealed that PGC-1α may play a critical role in skeletal muscle fiber type conversion by promoting fiber-type switching from glycolytic toward oxidative fibers [10]. The overexpression of PGC-1α under the control of the muscle creatine kinase (MCK) promoter induced a fiber type conversion, which is a characteristic of type I fibers, and prevented muscle mass wasting in transgenic mice subjected to denervation or fasting [11].

The therapeutic treatment of skeletal muscle atrophy with naturally occurring compounds has recently received increasing attention [12]. Pyrroloquinoline quinone (PQQ), a bacterially synthesized quinine, is a strong redox cofactor with multiple biological benefits including antioxidation, anti-cancer, anti-inflammation, the modulation of mitochondrial metabolism, and neuroprotection [13–16]. Gong et al. proposed that PQQ acts as an anti-hyperalgesic agent to inhibit the action of tumor necrosis factor alpha (TNF-α) and the formation of the lipid peroxide malondialdehyde in rats with chronic constriction injuries of the sciatic nerve [17]. Chowanadisa et al. elucidated that PQQ stimulated the phosphorylation of cAMP response element-binding protein (CREB) and further increased PGC-1α mRNA and protein expression. Treatment with PGC-1α siRNA attenuated mitochondrial biogenesis [18]. PQQ administration also modulates mitochondrial function to enhance lipid metabolism [19]. However, the effect of PQQ on PGC-1α and mitochondrial electron transport chain (ETC) complex expression in skeletal muscle following denervation has not yet been thoroughly investigated. Therefore, the purpose of the present study was to examine the potential of PQQ to protect against denervation-induced skeletal muscle atrophy.

In this work, we investigated the involvement of PGC-1α in sciatic nerve axotomy-mediated skeletal muscle wasting. Furthermore, we investigated whether the administration of PQQ prevented denervation-induced protein degradation. PGC-1α expression decreased during muscle wasting post denervation. Moreover, PGC-1α and mitochondrial ETC complexes were improved by treatment with PQQ, leading to the preservation of both type I and type II muscle fibers, accompanied by the alteration of mitochondrial OXPHOS bioenergetics.

## Materials and Methods

### Materials

ALZET® osmotic pumps (Model # 1004, 1.5 cm in length, 0.6 cm in diameter, 0.4 g weight, 28 day delivery at a pumping rate of 0.11 μl/h) were obtained from Durect Corporation (Cupertino, CA, USA). PQQ (Cat. No. 164–17083) was purchased from Wako Pure Chemical Industries, Ltd. (Japan). Other high-grade reagents and chemicals were purchased from local commercial companies.

### Animals and Treatments

C57BL6/J mice aged approximately 4–5 weeks and weighing approximately 22–24 grams were purchased from National Laboratory Animal Center (Hsinchu, Taiwan). All experimental procedures were approved by the Institutional Animal Care and Use Committee (Taipei Medical University, IACUC Approval No. LAC-2014-0243). The animals were randomly divided into 4 groups (n = 5–10 for each group): the control group (Con), a sham operation group (Sham), the denervation group (Den), and the PQQ-treated denervated group (Dep). All mice were housed under conditions of controlled temperature and humidity with a 12:12 h light:dark cycle and given standard chow diet and water ad libitum. Animals were anesthetized intraperitoneally with sodium pentobarbital (50 mg/kg body weight (bw)), and the right hindlimb of each mouse was denervated by removing an approximately 1 cm section of the sciatic nerve. The sham group was treated with the same operation without removing the sciatic nerve. During the operation, a PQQ-containing ALZET® osmotic pump (equivalent to 4.5 mg/day/kg bw for 28 days, diluted with normal saline solution) was implanted subcutaneously into the right lower abdomen of the mouse for the PQQ group. The skin wound was then closed with surgical clips coated with disinfectant (Premium Surgiclip^TM^, Covidien, Tyco Healthcare, Taiwan). The operation had no effect on the daily chow diet or water intake during the experimental period. The mice were sacrificed by cervical dislocation under deep isoflurane inhalation anesthesia on the appointed number of days following transection surgery. The gastrocnemius and soleus muscles were quickly excised from both hindlimbs, immediately immersed in cold isopentane, and stored at -80°C for subsequent analyses.

### Energy Metabolism Assay

At the end of the treatment period, the mice were sacrificed and the gastrocnemius muscle was excised and shredded with tweezers in a petri dish filled with basal Dulbecco’s Modified Eagle Medium (DMEM) without sodium bicarbonate adjusted to pH 7.4. After the sample was collected and dispersed into the XF24 islet capture microplate, 500 μl pre-warmed DMEM was added. After equilibrium was achieved, the basal state energy metabolism of the gastrocnemius muscle was analyzed by a Seahorse XF-24-Analyzer (Seahorse Bioscience, North Billerica, MA, USA). The program was set up as follows: 10 cycles of 3 min mixing, 2 min waiting, and 3 min measurement. The pH value, oxygen consumption rate (OCR, pmoles/min), and extracellular acidification rate (ECAR, mpH/min) were recorded simultaneously. The data were normalized to the protein content.

### Histochemical Assays and Fiber Size Measurements

Histopathological evaluation was performed on 10 μm frozen sections of muscle biopsy. Differential stains including Hematoxylin and Eosin (H & E), NADH-tetrazolium reductase (NADH-TR) and cytochrome *c* oxidase (COX) were performed according to previously described methods [20]. Finally, the slides were rinsed in deionized water and then mounted on coverslips for further microscope examination. Muscle fiber size was measured by Image J Software (NIH, Bethesda, MD, USA), and the values were normalized to the control group.

### Western Blot

Protein was isolated from the gastrocnemius muscle by homogenization in lysis buffer (PBS, 0.1% Triton X-100/1 mM EDTA, pH 7.4) containing complete protease inhibitors (Roche Diagnostics). Samples (20 μg) of total cell lysates were electrophoretically size fractionated by a 10% polyacrylamide sodium dodecyl sulfate–polyacrylamide gel and transferred onto a polyvinylidene difluoride membrane (PVDF) using the BioRad Mini Protean electrotransfer system. The blots were subsequently incubated with 5% skim milk in phosphate-buffered saline with Tween-20 for 1 h to block non-specific binding and probed overnight at 4°C with specific antibodies against PGC-1α, mitochondrial transcription factor A (TFAM), glyceraldehyde 3-phosphate dehydrogenase (GAPDH), and β-actin (Biochiefdom, Taipei, Taiwan), respectively. The membranes were then detected by incubation with appropriate peroxidase-conjugated secondary antibodies for 1 h at room temperature. The membranes were further washed with PBS containing Tween-20 (PBST) wash buffer. After the final PBST wash, signals were developed by enhanced chemiluminescence (Merck Millipore, Darmstadt, Germany) and monitored using the UVP BioSpectrum system (Analytik Jen. Upland, CA, USA). Signal intensity was quantified by Image J Software.

### Mitochondrial Electron Transport Chain Complex Assay

Following sacrifice, the gastrocnemius muscle was excised and minced with razor blades. The minced tissue was mixed with a 10-fold volume (W/V) of isolation buffer containing 100 mM KCl, 50 mM Tris-HCl, 2 mM EGTA, 0.5% bovine serum albumin (BSA), pH 7.4 at 4°C and homogenized in a mortar with 6 passes using a medium tight pestle. The homogenate was centrifuged at 2000 × g, 4°C, for 5 min. The supernatant was collected and centrifuged at 10,000 × g for 10 min. The supernatant was discarded and the pellet resuspended in isolation medium, and then centrifuged again at 10,000 × g for 10 min. The supernatant was discarded and the pellet resuspended in 500 μl of isolation medium. The protein concentration of the mitochondrial suspensions was determined using the bicinchoninic acid (BCA) kit with BSA as the standard (Pierce™ BCA Protein Assay Kit, Life Technologies, Taipei, Taiwan). For mitochondrial ETC complexes, 10% polyacrylamide gel electrophoresis was performed to separate proteins, followed by electroblotting onto PVDF membranes (Bio-Rad). The protein levels of mitochondrial marker proteins for oxidative phosphorylation (OXPHOS) complexes (20 kDa subunit of complex I, 30 kDa subunit of complex II, 48 kDa subunit of complex III, 40 kDa subunit I of complex IV and 55 kDa subunit α of complex V, F_0_F_1_ ATP synthase) were detected using the Rodent Total OXPHOS Complexes Western Blotting Antibody Cocktail according to the manufacturer's protocol (Abcam, Cambridge, UK).

### Messenger RNA Expression of Target Genes

Total RNA was extracted from the gastrocnemius muscle of both the denervated and control hindlimbs with TRIzol reagent (TriReagent, Cincinnati, OH, USA), based on the guanidine thiocyanate method. Frozen muscle was mechanically homogenized on ice in 1 ml of ice-cold TriReagent. Total RNA was solubilized in RNase-free H_2_O and quantified in duplicate by measuring the optical density (OD) at 260 nm. The purity of RNA was ensured by examining the OD260/OD280 ratio. Total RNA (2 μg) was synthesized into cDNA using the First Strand cDNA Synthesis Kit with the oligo (dT) primer method (Toyobo, Osaka, Japan) followed by DNAse treatment. A control reverse transcription (RT) reaction was performed in which the reverse transcriptase enzyme was omitted. The control RT reaction was used to ensure that the DNA did not contaminate the RNA sample. To measure the mRNA levels of target genes, quantitative RT-PCR was performed. After the synthesis of first-strand cDNAs from mRNAs, second-strand synthesis and amplification of target genes were performed as follows: the real-time PCR analysis of *PGC-1α*, *TFAM*, *UCP-2*, *cathepsin L (CPL)*, and *myosin heavy chain* (*MyHC*) *isoforms* (*MyHC Ib*, *MyHC IIa*, *MyHC IIb*, *MyHC IIx/d*) were performed in the presence of specific primers designed to amplify the target gene using the Ampliq master mix kit (Ampliqon, Skovlunde, Denmark) and the ABI thermal cycler system 7300 (Applied Biosystems, Foster City, CA, USA). The reaction mixture (25 μl) was subjected to 40 cycles of 30 sec at 96°C, 30 sec at 55–60°C, and 30 sec at 72°C and the results were analyzed using ABI 7300 system software.

### Statistical Analysis

All data are reported as the mean ± standard error of the mean (SEM). A one-way ANOVA with Fisher's post hoc test for least squares differences was used to evaluate the differences between the Con, Sham, Den and Dep groups at different time points. Statistical significance is defined as *P*< 0.05.

## Results

### PQQ Administration Retards Muscle Atrophy and Restores Mitochondrial Function

**Fig 1:**
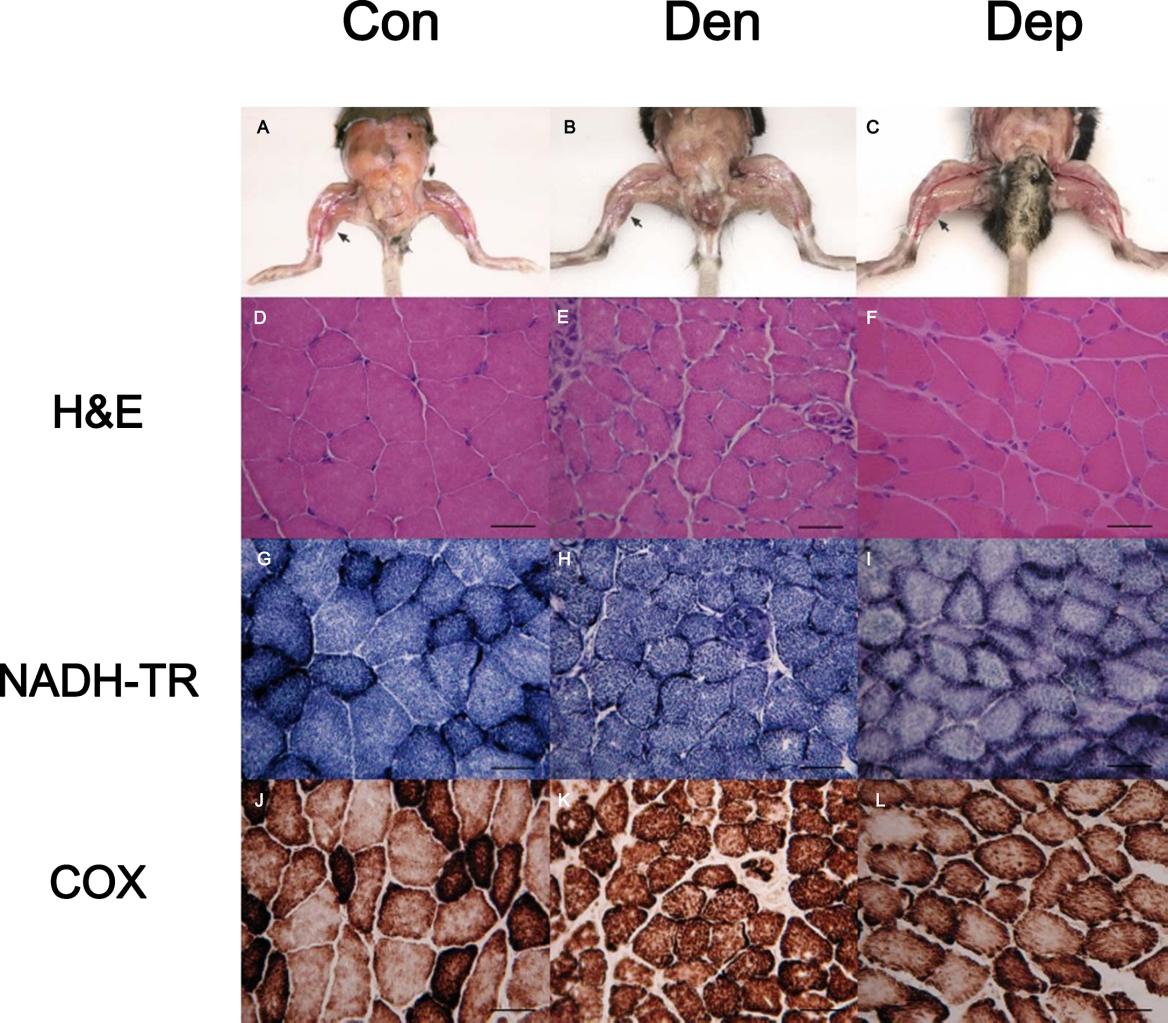
Histochemical staining of the gastrocnemius muscle post denervation. H & E, NADH-TR, and COX stains were performed on 10 μm thick transverse frozen muscle section sections of gastrocnemius muscles from the control (Con), denervated (Den), and PQQ-treated denervated groups (Dep) on the 21^st^ day post denervation. Scale bar: 20 μm; 200 ×. (A-C, arrows point to the hindlimb muscle; D-F, H & E stain; G-I, NADH-TR stain; J-L, COX stain).

To evaluate the effects of axotomy on skeletal muscle wasting, C57BL6/J mice were unilaterally denervated by severing the sciatic nerve of the right hindlimb, with the left limb serving as the internal control. In contrast to the control group, the 21^st^ day post-denervation resulted in muscle atrophy and a shift towards oxidative fibers, as evidenced by the reduced fiber cross-sectional areas of H&E staining and the higher intensity of NADH-TR and COX staining (Fig 1E, 1H and 1K). NADH-TR staining demonstrated that the fibers with mosaic patterns, predominantly fast-twitch fibers, occupied the largest area in the control group, but almost all fibers were slow-twitch like fibers after denervation. To investigate whether PQQ protects against muscle atrophy induced by denervation, the PQQ-containing ALZET® osmotic pump was implanted subcutaneously into the right lower abdomen at the time of the denervation operation, and the wound was sutured. Continuous supplementation with PQQ delayed post-denervation muscle wasting and resulted in the partial preservation of the content of visibly larger fibers (Fig 1F, 1I and 1L). Furthermore, a similar mosaic pattern was restored in the treated group, matching that in the control group (Fig 1I).

### Decrease in PGC-1α-Related Gene Expression and Fiber Size following Denervation

**Fig 2:**
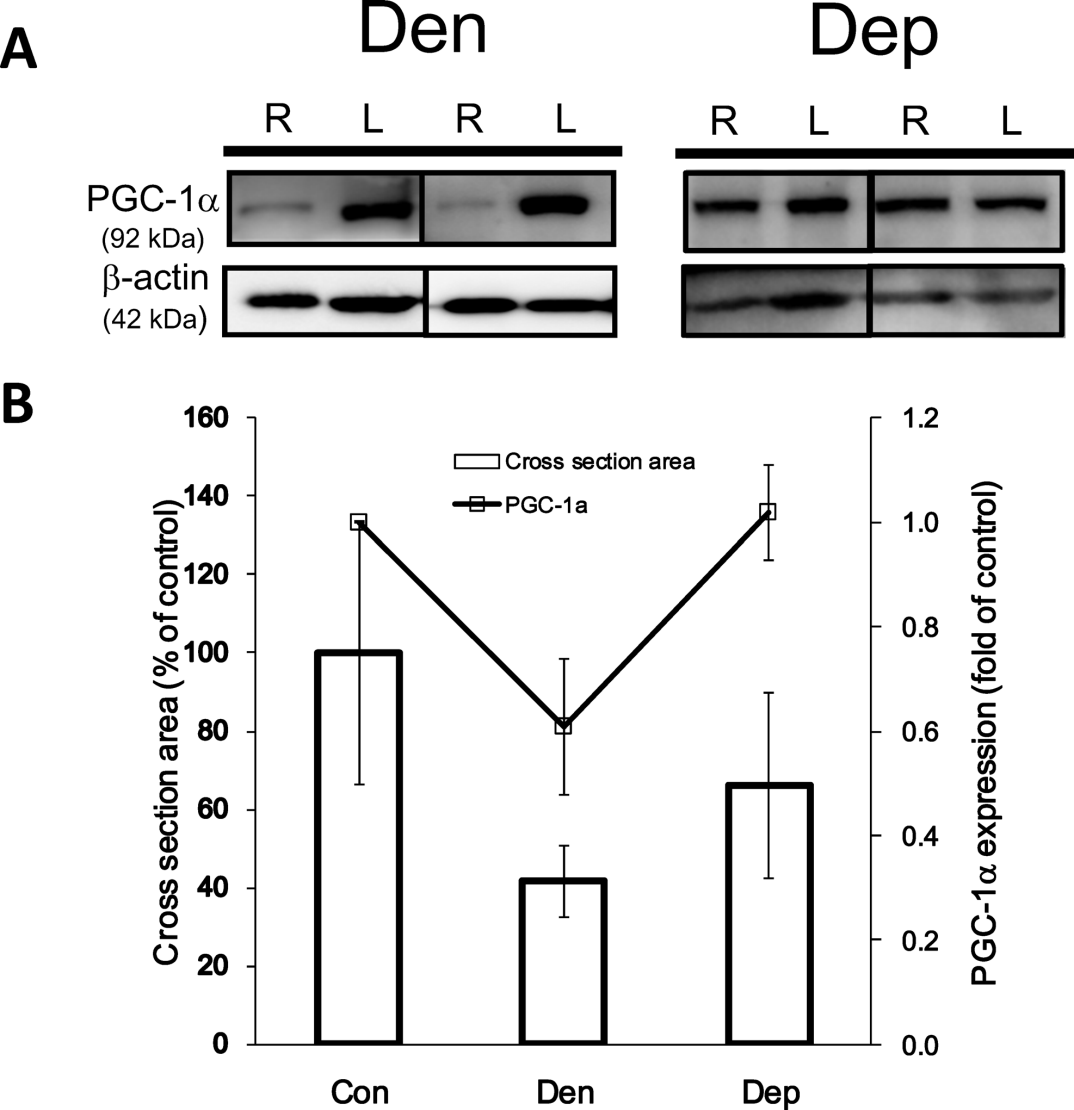
Decreased PGC-1α level in the gastrocnemius muscle following denervation and recovery by PQQ treatment. (A) The PGC-1α protein level was determined by western blot analysis of the gastrocnemius muscles of the denervated (Den) and PQQ-treated denervated (Dep) groups. β-actin is defined as the housekeeping protein and used as a loading control. (B) Statistical analysis of the data from two independent experiments (n = 4 to 5 per group). # and *, *P*<0.05 indicating a significant difference compared to the Con and Den groups, respectively.

We further investigated the role of PGC-1α in denervation induced muscle atrophy. As shown in S1 Fig, at the timepoints studied here, the mRNA expression of *PGC-1α*, *UCP-2*, *TFAM*, and *CPL* in the skeletal muscle were enhanced and then decreased on the 3^rd^ and 21^st^ day post-denervation, respectively. Similarly, the protein levels of PGC-1α on the 21^st^ day after denervation were significantly (*P*<0.05) reduced (Fig 2A), which was accompanied by a dramatic reduction in the myofibril cross sectional area (Fig 2B). Furthermore, PQQ administration resulted in a significant restoration in the level of the PGC-1α protein in the denervated right hindlimb skeletal muscle as normalized to the left hindlimb muscle. Meanwhile, the average fiber size was attenuated to approximately 40% of the cross-sectional area compared to the control group.

### PQQ Normalizes Energy Metabolism following Denervation-Induced Perturbations

**Fig 3:**
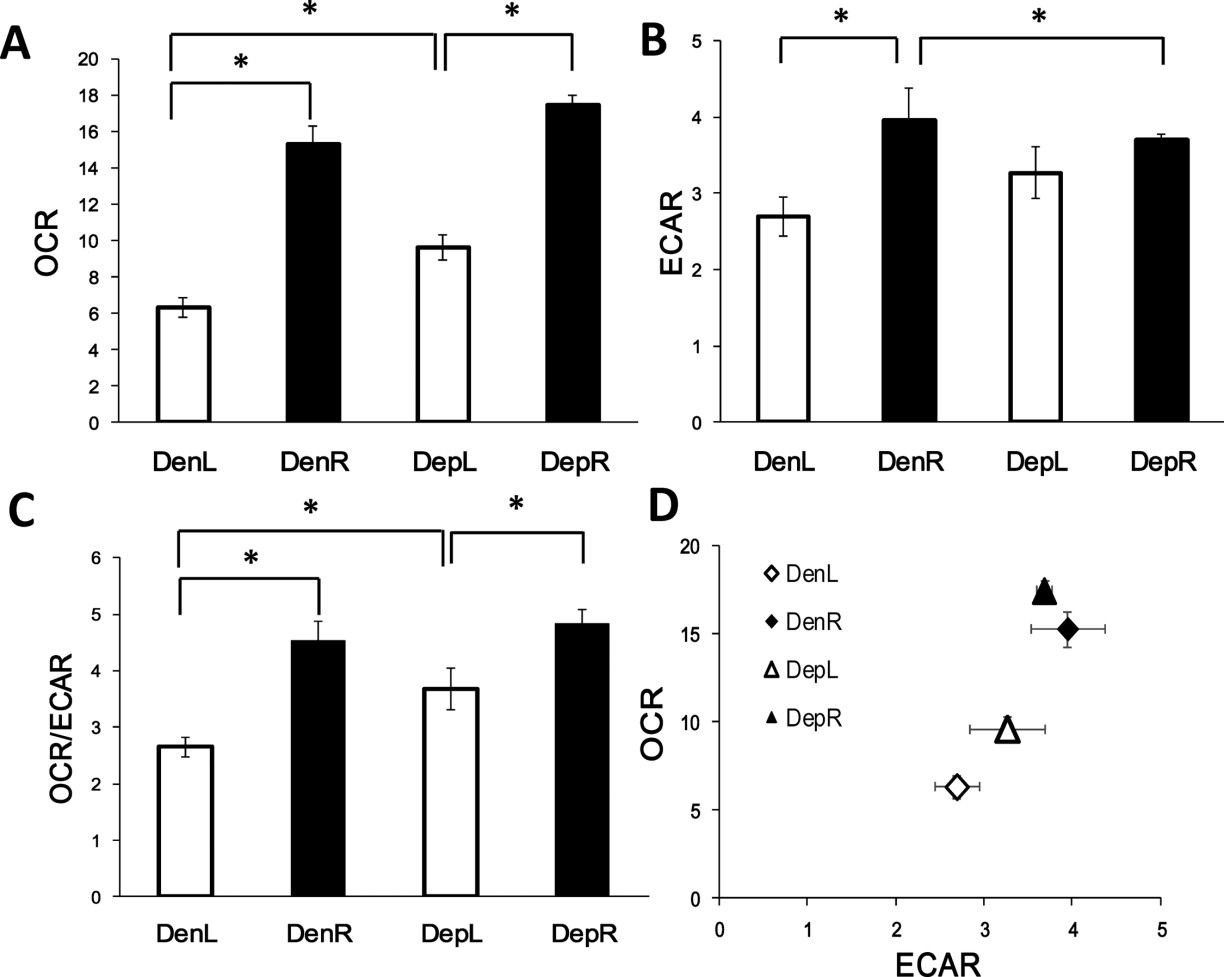
Effect of PQQ on energy metabolism in denervated gastrocnemius muscle. On the 21^st^ day after denervation, the gastrocnemius muscle was excised to determine basal OCR (A), ECAR (B), and the OCR to ECAR ratio (C). (D) The OCR and ECAR values of Den and Dep were plotted to show the difference in the metabolic profile between these groups. Values (n = 3 to 5 per group) represent means ± SEM. *, *P*<0.05, indicating a significant difference between groups. DenL, DenR, DepL, and DepR represent the left internal control (L) and the right denervated (R) hindlimb muscle from the denervated (Den) or PQQ-treated denervated group (Dep), respectively.

To determine how PQQ influences the effects of denervation on muscle metabolism, the basal OCA (mitochondrial activity), ECAR (glycolytic rate), and the ratio of OCR to ECAR were examined. We found that both basal OCR and ECAR were significantly (*P*<0.05) increased in the control right hindlimb skeletal muscles compared to the left hindlimb muscles (Fig 3A and 3B). Comparing the left internal control hindlimb muscles, the PQQ-treated group exhibited higher OCR and ECAR than the denervation group alone. The PQQ-treated denervated group had a higher OCR/ECAR ratio than the denervated group (Fig 3C). This demonstrated that the energy metabolic profile of the PQQ-treated denervated group was reprogrammed toward mitochondrial oxidative phosphorylation (Fig 3D).

### Changes in Muscle Fiber Type following Denervation

**Fig 4:**
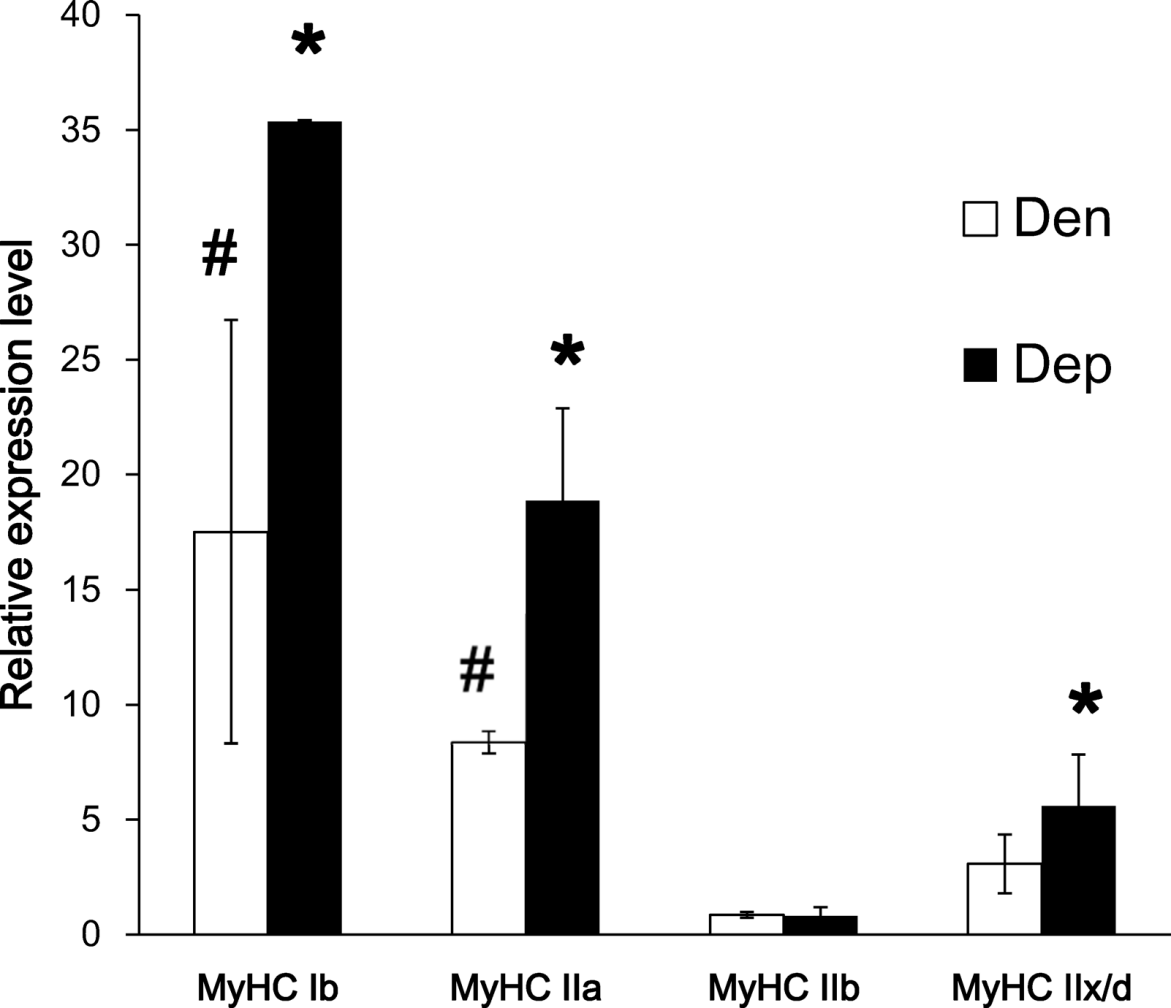
mRNA expression of myosin following denervation. Quantitative real-time RT-PCR for myosin subtypes of the gastrocnemius muscle in the denervated (Den) and PQQ administered groups (Dep) on the 21^st^ day after denervation. Data are expressed as the right denervated muscle relative to the contralateral non-denervated left hindlimb muscle of the same mouse, normalized to control group. # and *, *P*<0.05, indicating significant differences compared to the Con (control) and Den (denervation) groups, respectively.

We further examined whether PQQ treatment affects the change in muscle fiber type post denervation. As shown in Fig 4, the expression levels of MyHC subtypes in the gastrocnemius muscle revealed that denervation induced a significant shift in muscle fibers towards type Ib and IIa compared with the control group (*P*<0.05). Interestingly, PQQ administration not only enhanced type Ib and IIa fiber expression but also induced type IIx/d fiber expression.

### Changes in Glycolytic and Mitochondrial Energy Metabolism following Denervation

**Fig 5:**
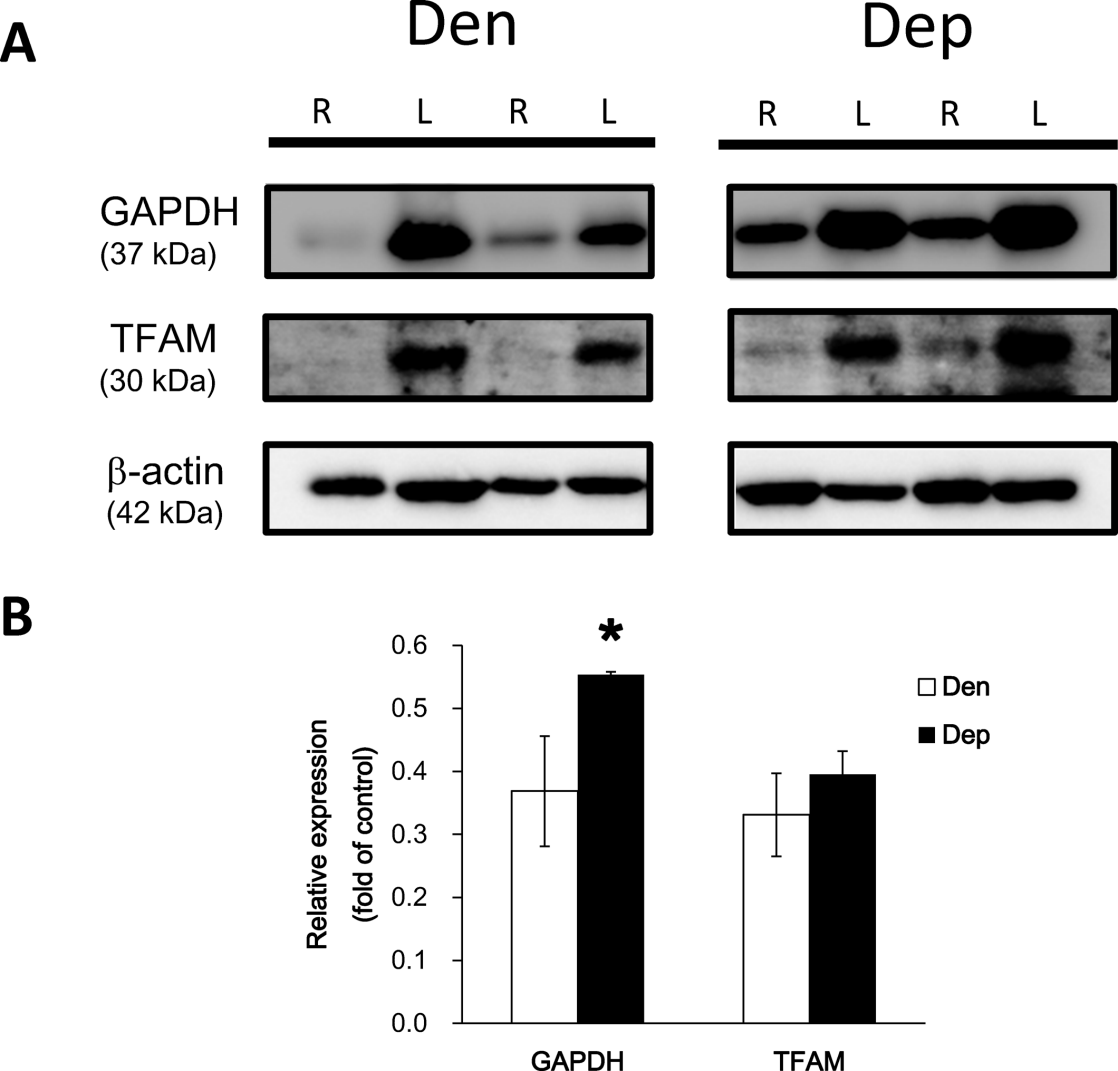
Changes in glycolytic and mitochondrial energy metabolism following denervation. (A) Western blot analysis of GAPDH and TFAM expression in the denervation (Den) and PQQ-administered denervation groups (Dep). (B) Statistical analysis of western blot data (n = 3 to 5 of each). *, *P*<0.05, indicating a significant difference compared to the Den group.

We further investigated proteins involved in the regulation of energy metabolism post denervation. As shown in Fig 5A, the protein level of GAPDH, one of the critical proteins involved in glycolysis, was significantly decreased in the right hindlimb with sciatic axotomy (*P*<0.05). Furthermore, TFAM expression in the right hindlimb was also lower than that in the control left hindlimb muscle after denervation. However, continuous administration of PQQ dramatically enhanced the levels of both GAPDH and TFAM (Fig 5B).

### Recovery of the Function of Mitochondrial Complexes after PQQ Treatment

**Fig 6:**
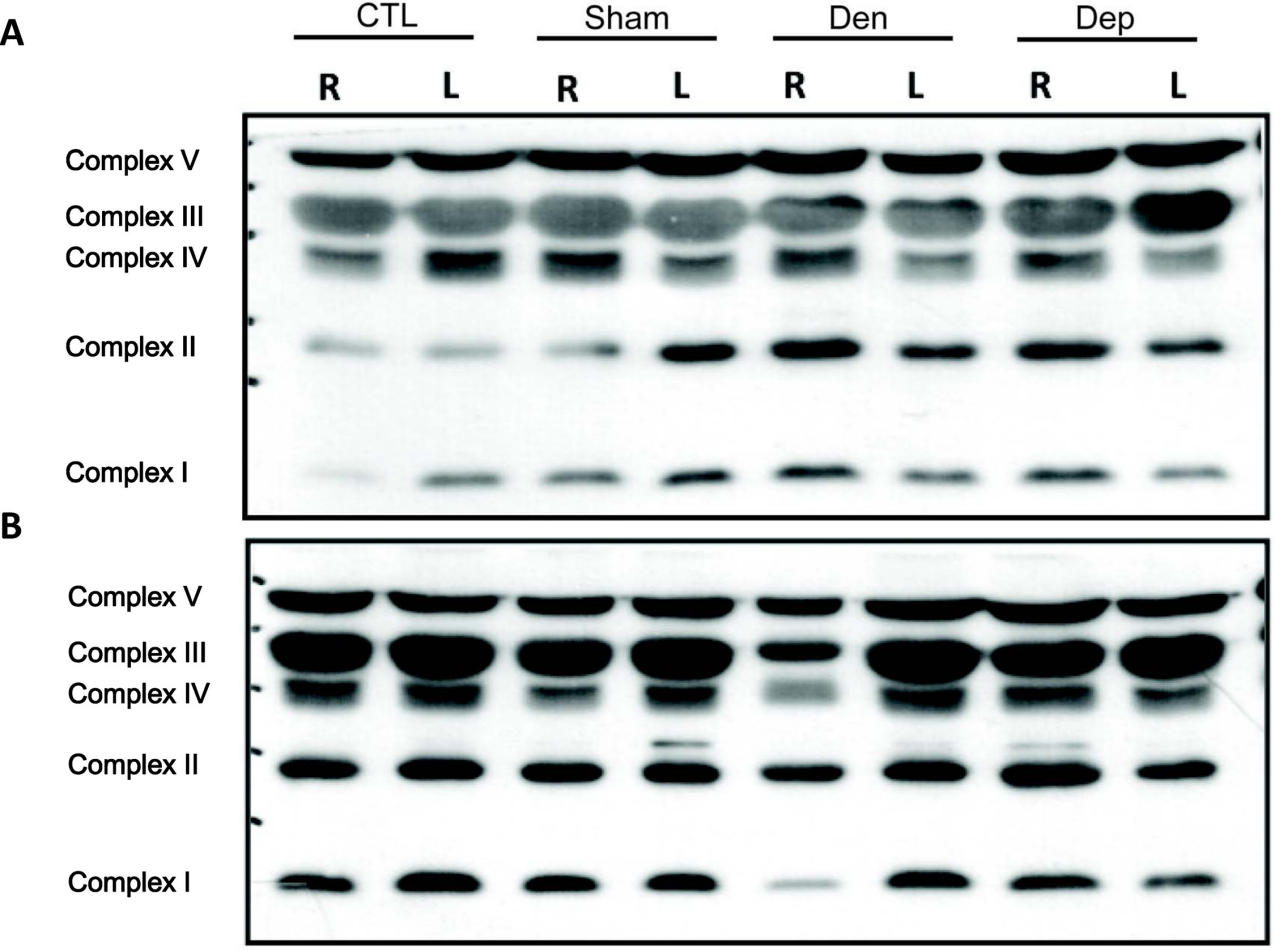
Western blot of OXPHOS mitochondrial complexes. An antibody cocktail against proteins representing the five mitochondrial oxidative phosphorylation complexes was used to examine the expression of mitochondrial proteins in skeletal muscle from the control (Con), sham-operated (Sham), denervated (Den), and PQQ administered groups (Dep) on the 7^th^ (A) and 21^st^ days (B) after denervation.

We further examined whether denervation affects mitochondrial oxidative function through the modulation of mitochondrial ETC complexes. The expression of mitochondrial complexes in skeletal muscle from the control, sham, denervated, and PQQ administered groups were evaluated on the 7^th^ and the 21^st^ days post-denervation, respectively. The OXPHOS complexes exhibited no change on the 7^th^ day after denervation (Fig 6A). However, the protein levels of all of the OXPHOS complexes except for complex V were significantly (*P*<0.05) decreased in the right hindlimb on the 21^st^ day after denervation (Fig 6B). The most dramatic decrease was observed in NADH-TR and COX. However, the protein levels of complex II and IV subunits were significantly restored upon PQQ treatment (*P*<0.05, S2 Fig).

## Discussion

Traumatic peripheral nerve injury is complicated due to the irreversible effects if re-innervation is delayed. The absence of motor recovery after nerve damage may result from a failure of synapse reformation after prolonged denervation rather than a failure of axonal growth [21]. The other complicating factor may be the denervation of skeletal muscle, induced by apoptosis and muscle atrophy [22, 23]. Many cellular structural alterations have been described after denervation, including changes in the number and size of mitochondria [24]. During the first 4 months after nerve transection, the loss of capillaries was found to be nearly linear, with a gradual week by week decrease [25]. Previous histochemical study showed that the number of type II fibers observed in frozen muscle section was reduced by about forty percent four weeks following denervation, although this finding was not statistically significant. Marked changes revealed that the percentage of slow myosins remained low in the contralateral gastrocnemius, whereas it increased to 95% in the denervated gastrocnemius [23, 26]. Therefore, fiber type shifting was observed after denervation of the sciatica nerve. These results mentioned above were compatible with our findings that the number of glycolytic fibers decreased after denervation, as evidenced by the reduced protein level of GAPDH in the gastrocnemius muscle (Fig 1H and 1K and Fig 4).

To our knowledge, this is the first *in vivo* study to examine the effect of the naturally occurring antioxidant PQQ on denervation-induced skeletal muscle atrophy. Continuous and regular administration of PQQ was achieved using an ALZET® osmotic pump, which has proven to be an extremely useful tool for the long-term, continuous delivery of compounds for research purposes [27, 28]. We examined the gastrocnemius rather than the soleus muscle, as almost half of the gastrocnemius muscle quickly wasted away 10 days post denervation [29]. Histoenzymology of skeletal muscles has been successfully applied to classify functionally distinct fiber groups. In our study, short-term denervated gastrocnemius muscle exhibited a more oxidative phenotype, as shown by the greater content of NADH-TR and COX activities and a shift to type Ib and IIa muscle fibers (Figs 1 and 4) compared with control muscle. Continuous supplementation of PQQ resulted in a partial restoration of fiber size and mitochondrial function. Many studies have proposed possible mechanisms by which PGC-1α regulation leads to the fiber type switch. When PGC-1α is expressed at physiological levels in transgenic mice driven by an MCK promoter, a fiber type conversion is observed. The expression of PGC-1α in skeletal muscle may be regulated by calcium/calmodulin kinase IV and calcineurin A activity through the binding of the CREB protein to the PGC-1α promoter [30], myocyte enhancer factor 2 and forkhead in rhabdomyosarcoma. Myogenic basic helix-loop-helix proteins, especially MyoD, could activate PGC-1α expression by targeting its promoter. Furthermore, the lack of PGC-1α leads to decreased mitochondrial content, reduced muscle mass, and a myopathic phenotype as evidenced by the accumulation of multivesicular bodies and tubular aggregates [31].

Mitochondrial dysfunction is one of the most frequently critical mechanisms of muscle atrophy. Recently, one study showed that caloric restriction (CR) attenuated age-related muscle loss through activating AMPK and SIRT1, leading to an increase in COX IV content. CR induced the reprogramming of glycolysis, and mitochondrial oxidative phosphorylation was accompanied by the maintenance of muscle mass during aging [32]. Furthermore, a clinical study revealed that long-term denervation induced human skeletal muscle atrophy is accompanied by decreases in glycogenolytic and glycolytic enzymatic activities [33]. Interestingly, Singh and Hood showed that oxygen consumption and ROS production were unexpectedly up-regulated in the subsarcolemmal mitochondria isolated from the denervated tibialis anterior muscles [8]. In the present study, we found that the denervated group showed some significant phenomena: (1) a decrease in the protein level of GAPDH and (2) increases in the OCR and ECAR values on the 21^st^ day after denervation. Because of the complexity of the rate of mitochondrial dysfunction and muscle atrophy during different periods of denervation, PQQ treatment retarded the execution of denervation-induced muscle atrophy, perhaps by modulating the early adaptations in mitochondrial function and metabolic behavior. This event may also echo the findings that PGC-1α plays a central role in regulating the switch of fibers from the glycolytic towards the oxidative type [10]. In the event of denervation, the degradation of PGC-1α and ETC complexes and the decrease in the glycolytic enzymatic activity result in the up-regulation of mitochondrial phosphorylation and glycolysis in response to the energy leakage. However, sustained accumulation of ROS finally leads to the activation of proteolytic signals including caspases, apoptosis, autophagy, and the proteasome system, all of which promote protein degradation. Similar results were revealed in the skeletal muscle cell model, which showed that the OXPHOS levels are significantly increased in galactose-treated cells compared with glucose-treated cells and that the ECAR value is up-regulated when ATP synthesis is blocked [34].

Our results showed a transient increase in the PGC-1α mRNA level after short-term denervation (S1 Fig), but the overall PGC-1α protein level decreased (Fig 2). Consistent with previous observations of expression [35], on the 3^rd^ day post-denervation, the transient increase in the embryonic-type nicotinic acetylcholine receptor (nAChR) is in marked contrast to the expression of the adult-type subunit ε-RNA; however, long-term muscle denervation finally resulted in the reduced expression of embryonic-type nAChRs. Thus, we proposed that the trend in the expression of PGC-1α and its regulated targets is similar to the trend of the expression of nAChR in adaptation to denervation. An action is completed when signals are transmitted from the brain to the skeletal muscle. An action potential transmits the message to the skeletal muscles to contract via nAChRs. After denervation, mitochondria have been noted to play an important apoptotic role, acting as a central modulator [36]. CPL is a lysosomal proteinase whose expression is also up-regulated in skeletal muscle atrophy resulting from disuse and denervation [37]. Rapid disuse and denervation atrophy associated transcriptional changes may be similar to those of muscle wasting during systemic diseases. It has also been reported that metabolic stress signaling-induced mitochondrial membrane potential disruption is accompanied by an increase in the cytoplasmic free calcium (Ca^2+^) concentration [38]. CPL was finally activated followed by the induction of the Ca^2+^-dependent protein kinase C [37]. In the present study, we found that the CPL mRNA level significantly increased compared with the control and sham groups on the 3^rd^ day post-denervation (S1 Fig). This suggests that CPL may have a role in association with PGC-1α in the process of protein degradation after denervation.

**Fig 7:**
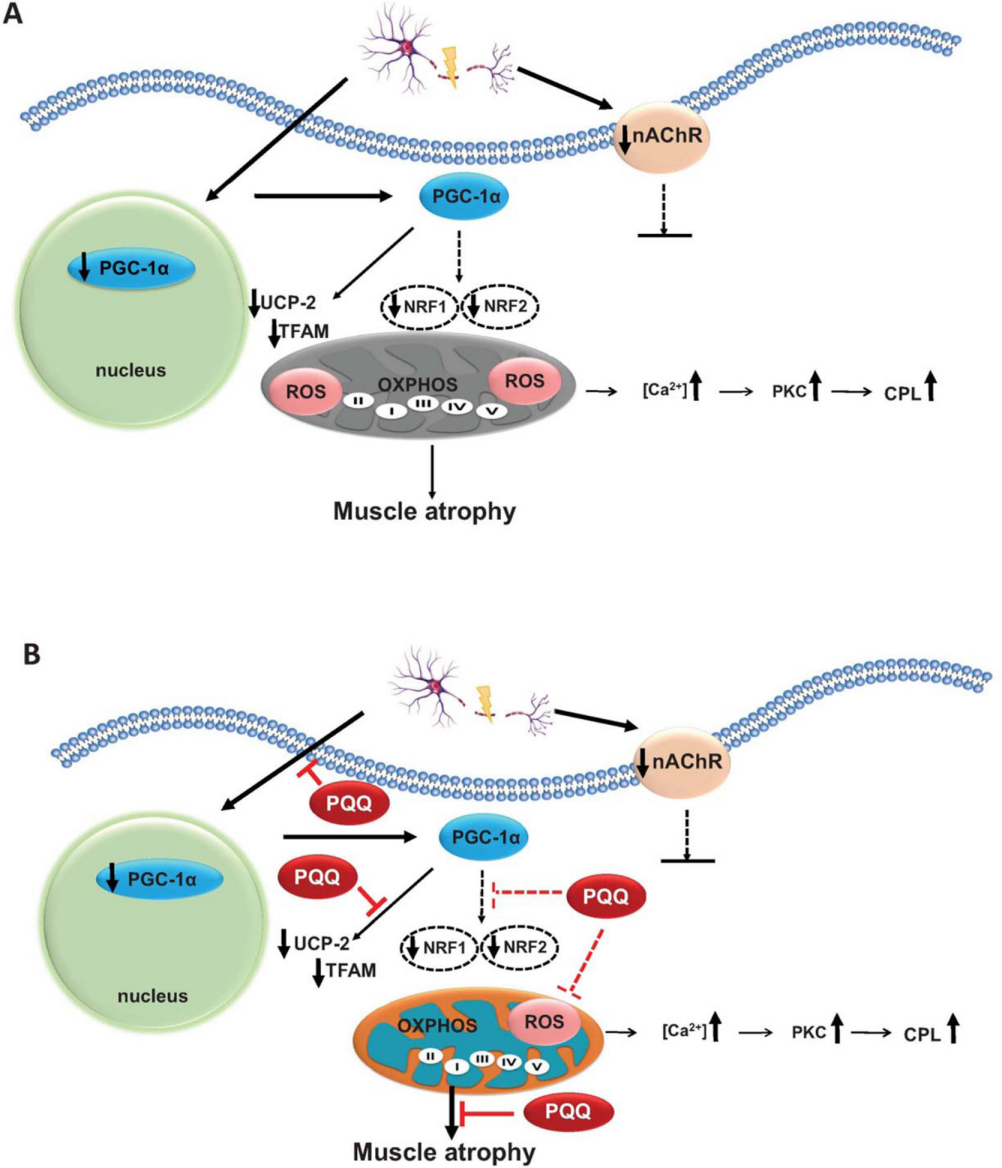
Summary of sciatic denervation-stimulated signaling transduction leading to skeletal muscle twitch and atrophy, and the possible actions of PQQ. In the event of denervation, PGC-1α is attenuated and subsequently UCP-2, TFAM, NRF1 and NRF2 are down-regulated. Thereafter, mitochondrial membrane potential disruption and up-regulation of CPL and PKC occur due to the accumulation of ROS and the increase in [Ca^2+^], leading to the protein degradation and muscle atrophy (A). Administration of PQQ results in an increase in the PGC-1α protein level, slowing protein degradation and muscle atrophy after denervation. Eventually, the strong antioxidant PQQ has the effect of reprogramming the mitochondrial OXPHOS integrity and metabolic bioenergetics (B).

PQQ is the third bacterial redox cofactor after nicotinamide and flavin, and it has been shown to be an essential nutrient for animal growth. PQQ not only serves to mediate redox reactions in the mitochondrial respiratory chain but also plays a potential role in scavenging ROS and attenuating oxidative stress in the mitochondria [39]. Chowanadisai et al. demonstrated that PQQ phosphorylates CREB, which subsequently activates the promoter of PGC-1α and increases PGC-1α mRNA expression [18]. Furthermore, the nuclear respiratory factors NRF-1, NRF-2, and TFAM are activated. Thus, PQQ can induce mitochondrial biogenesis in mouse hepatocytes. However, few studies have investigated whether OXPHOS complexes are affected. We found that complex function (particularly complex II and IV) is affected following denervation (S2 Fig). However, PQQ (the activator of PGC-1α) was able to restore the function of OXPHOS complexes in denervated skeletal muscles. Overall, the possible mechanism involved in PQQ-related retardation against denervation-induced skeletal muscle atrophy is illustrated in Fig 7.

## Conclusions

From a clinical perspective, multiple pathological changes are involved such as axon degeneration, the withdrawal of Schwann cells from the region of the neuromuscular junction, and muscle atrophy. Denervation of the muscle results in myofiber atrophy, fibrosis, and fatty tissue infiltration. These changes become irreversible when innervation is delayed. Thus, it is important to prevent skeletal muscle atrophy to avoid ongoing apoptosis. Taken together, this work demonstrates the critical role of PGC-1α in myopathogenesis. To prevent atrophy, activation of the mitochondrial OXPHOS pathway via the PGC-1α activator may provide an additional avenue for novel treatments in the early stages of muscle denervation.

## Supporting Information

S1 Fig mRNA Expression of PGC-1α and PGC-1α-regulated factors after denervation. Quantitative real-time RT-PCR for *PGC-1α*, *UCP-2*, *TFAM*, and *CPL* in the control (Con), sham-operated (Sham) and denervated (Den) gastrocnemius muscles on the 3^rd^ day and 21^st^ days after transection surgery. Data are expressed as the relative fold, representing the right denervated hindlimb muscle compared to the contralateral non-denervated left hindlimb muscle of the same mouse. *PGC-1α*, *UCP-2*, *TFAM*, and *CPL* were significantly increased on the 3^rd^ day, and then gradually decreased on the 21^st^ day after denervation (n = 3–5 for each group). *, *P*<0.05 compared with other groups.
(JPG)

S2 Fig Quantitation of Western blot data from OXPHOS mitochondrial electron transport chain complexes. The expression of five mitochondrial ETC complexes varied in response to various treatments. # and *, *P*<0.05, indicating a significant difference compared to the control (Con) and denervation (Den) groups, respectively.
(JPG)

## Abbreviations

CPL: Cathepsin L
COX: cytochrome *c* oxidase
CREB: cAMP response element-binding protein
ETC: electron transport chain
MyHC: myosin heavy chain
NADH-TR: NADH tetrazolium reductase
PQQ: Pyrroloquinoline quinone
PGC-1α: peroxisome proliferator co-activator 1 alpha
TFAM: mitochondrial transcription factor A
UCP-2: uncoupling protein-2
