# Supplementary figures and images for: Pyrroloquinoline Quinone Resists Denervation-Induced Skeletal Muscle Atrophy by Activating PGC-1α and Integrating Mitochondrial Electron Transport Chain Complexes
Source: PLoS One. 2015 Dec 8;10(12):e0143600. doi: 10.1371/journal.pone.0143600 (PMC4672922; doi:10.1371/journal.pone.0143600)

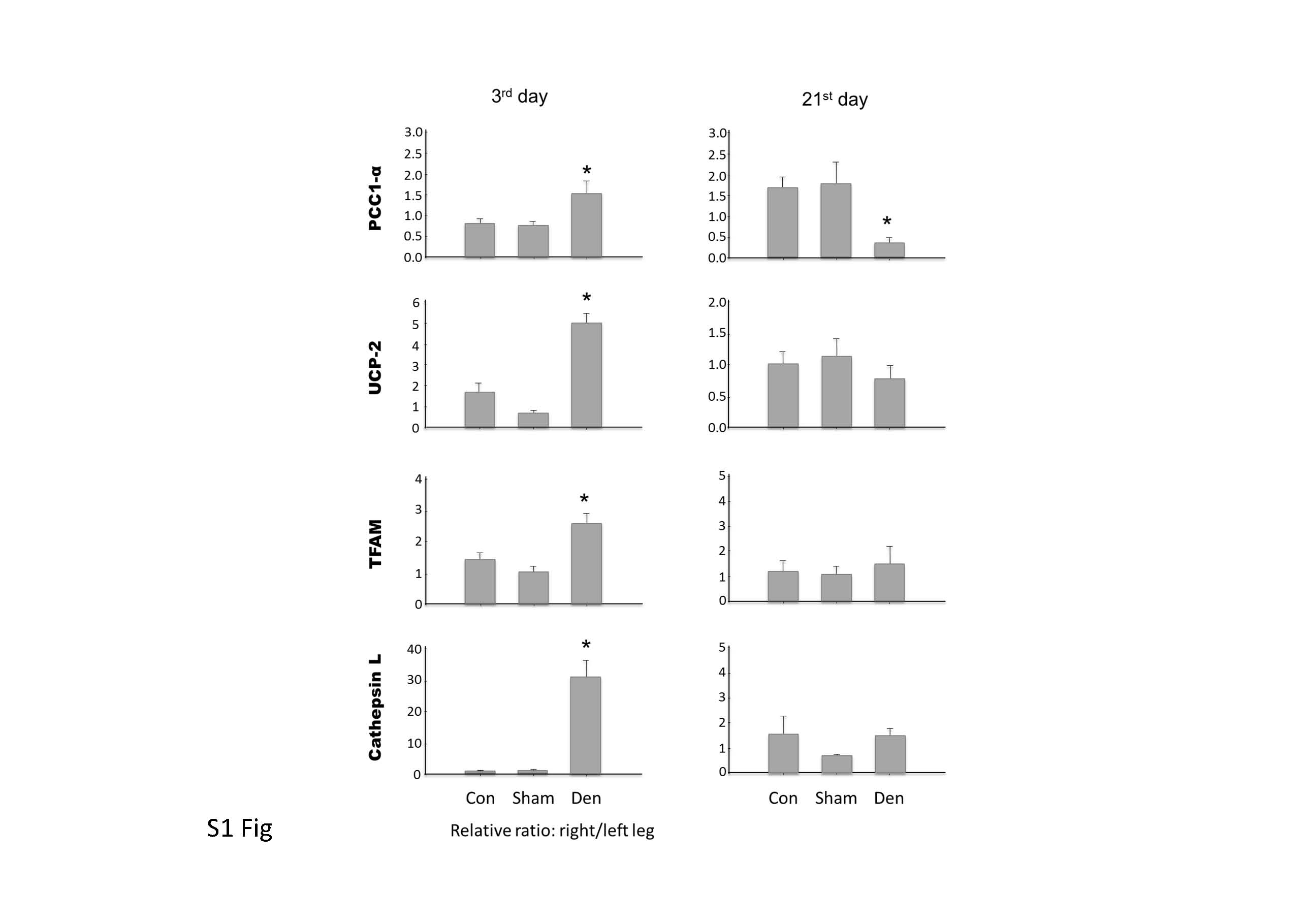

Supplement: S1 Fig — Quantitative real-time RT-PCR for PGC-1α, UCP-2, TFAM, and CPL in the control (Con), sham-operated (Sham) and denervated (Den) gastrocnemius muscles on the 3rd day and 21st days after transection surgery. Data are expressed as the relative fold, representing the right denervated hindlimb muscle compared to the contralateral non-denervated left hindlimb muscle of the same mouse. PGC-1α, UCP-2, TFAM, and CPL were significantly increased on the 3rd day, and then gradually decreased on the 21st day after denervation (n = 3–5 for each group). *, P<0.05 compared with other groups. (JPG) [file pone.0143600.s001.jpg]

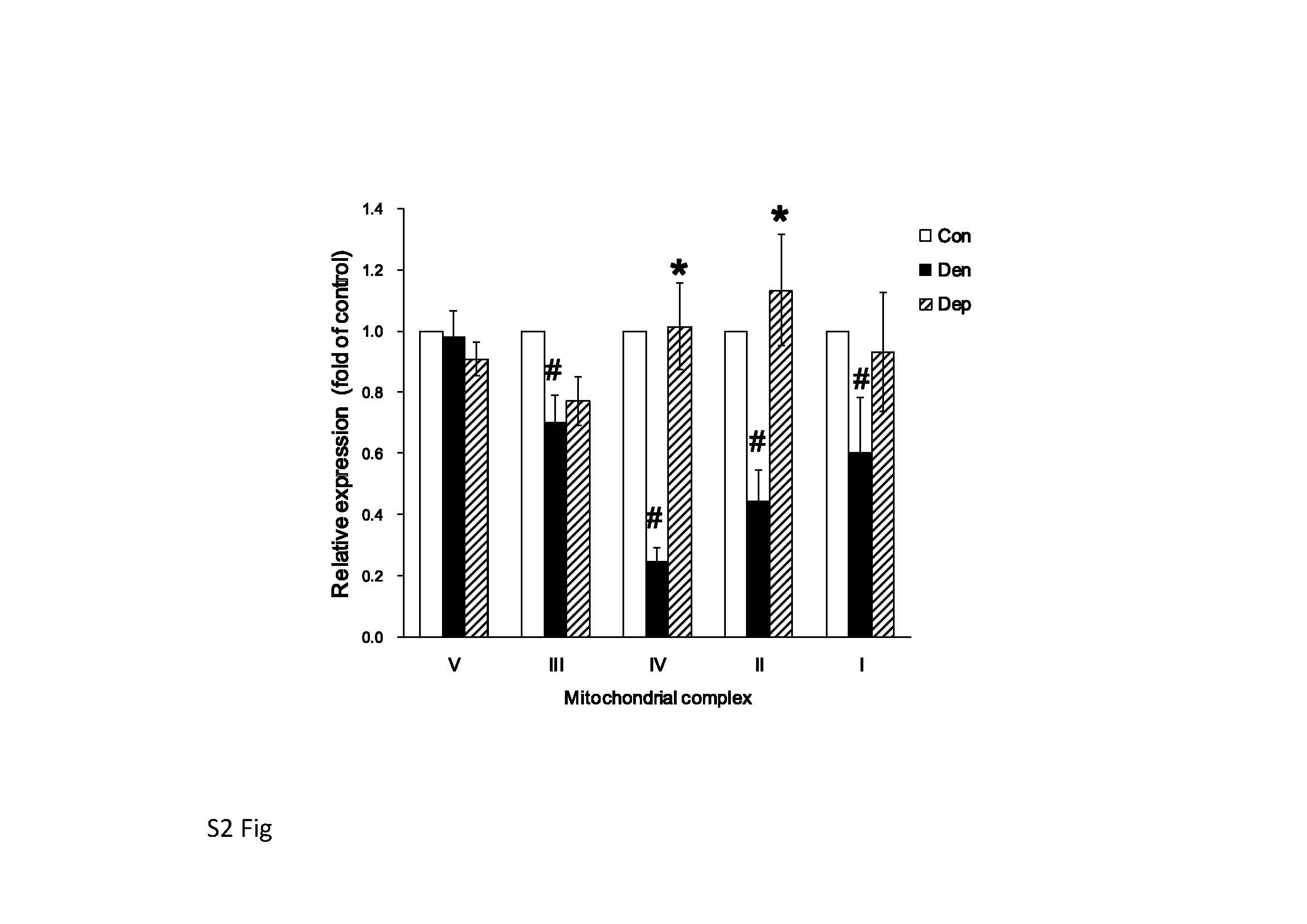

Supplement: S2 Fig — The expression of five mitochondrial ETC complexes varied in response to various treatments. # and *, P<0.05, indicating a significant difference compared to the control (Con) and denervation (Den) groups, respectively. (JPG) [file pone.0143600.s002.jpg]
